# Supplementary material for: Subpopulations of sensorless bacteria drive fitness in fluctuating environments
Source: PLoS Biol. 2020 Dec 3;18(12):e3000952. doi: 10.1371/journal.pbio.3000952 (PMC7738171; doi:10.1371/journal.pbio.3000952)
Supplement: S1 Table — Experiments that have been discarded from further analysis are grayed out. For experiments acquired before November 2015, data are taken from [6]. (PDF) [file pbio.3000952.s001.pdf]

condition	date	# growth channels	# full cell cycles	# observations	# cells at switch	# estimated lags	# arrested cells at switch
mg1655	20150812	6	364	17283	NA	NA	NA
glucose	20150616	31	2021	92270	NA	NA	NA
glucose	20150617	30	1920	91276	NA	NA	NA
lactose	20150624	30	1649	86776	NA	NA	NA
lactose	20150630	30	1671	85949	NA	NA	NA
memory 4h	20150703	28	1431	76295	462	445	3
memory 4h	20150708	30	1600	88410	536	511	6
memory 6h	20151204	30	1848	107195	548	524	10
memory 8h	*20151218*	*29*	*1821*	*115062*	*NA*	*NA*	*NA*
memory 8h	20180206	31	NA	66894	332	312	1
memory 12h	20180207	30	1517	84568	353	340	3
memory 12h	20180216	30	1633	82626	344	329	4
memory 12h (old)	*20160526*	*31*	*1178*	*83954*	*NA*	*NA*	*NA*
memory 16h	20160912	39	2437	134488	444	419	8
memory 20h	20161014	34	2224	133238	382	355	10
memory 24h	20161007	31	2343	135928	350	334	6
memory 24h	*20180313*	*9*	*57*	*9192*	*NA*	*NA*	*NA*
low [lactose]	20190614	24	580	27973	115	105	1
low [lactose]	20190605	38	528	25028	193	178	1
late	*20161021*	*22*	*865*	*83093*	*NA*	*NA*	*NA*
late	*20170108*	*30*	*1789*	*103600*	*NA*	*NA*	*NA*
late	20180516	30	2370	104792	166	155	0
late	*20180615*	*10*	*794*	*34532*	*NA*	*NA*	*NA*
ramp40min	20171121	30	371	29252	164	154	1
ramp40min	20180319	32	496	33684	195	185	1
preIPTG5uM	20180514	31	385	28585	178	167	0
preIPTG5uM	20180531	29	410	26780	170	161	0
lacIoe	20180116	31	796	55447	177	161	0
lacIoe	*20180123*	*29*	*758*	*49517*	*NA*	*NA*	*NA*
lacIoe	20180214	31	761	46967	157	142	1
lacIoe preIPTG10uM	20180604	33	410	28272	174	160	1
glyc to lac	20170919	26	139	28832	137	123	NA
glyc to lac	20170920	29	NA	32475	142	132	NA
glcLac lac	20171114	31	842	47798	180	171	0
glcLac lac	20180108	31	804	50630	174	170	4
glcLac lac	*20180606*	*9*	*579*	*25851*	*NA*	*NA*	*NA*
