# Supplementary figures and images for: Subpopulations of sensorless bacteria drive fitness in fluctuating environments
Source: PLoS Biol. 2020 Dec 3;18(12):e3000952. doi: 10.1371/journal.pbio.3000952 (PMC7738171; doi:10.1371/journal.pbio.3000952)

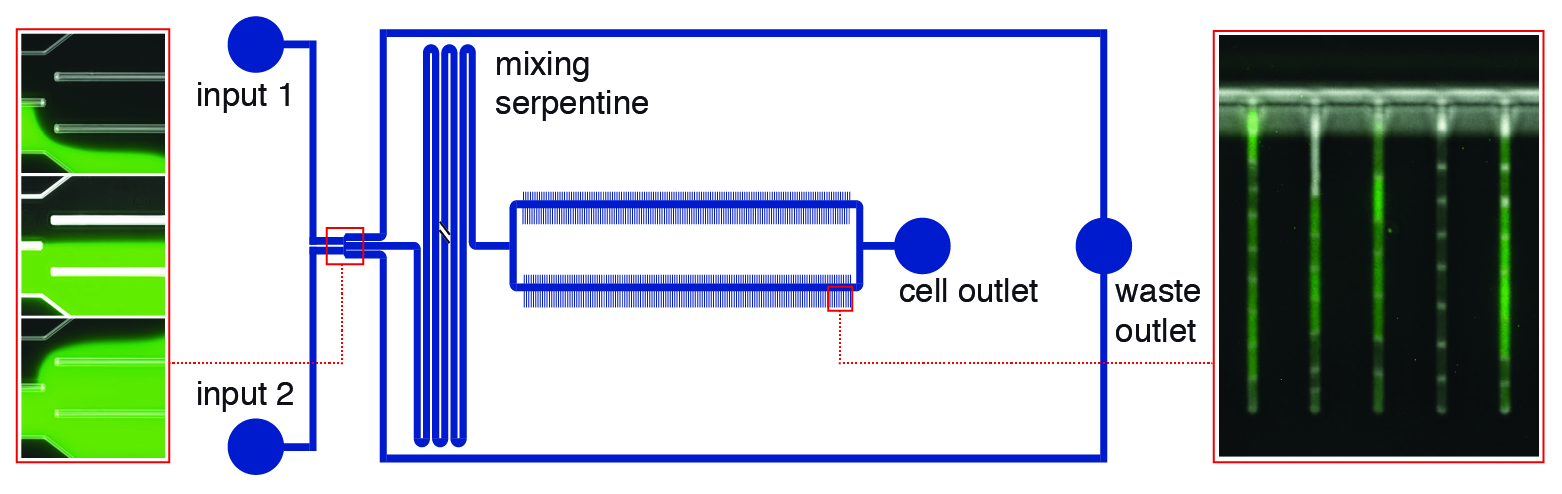

Supplement: S1 Fig — The DIMM combines a dial-a-wave mixing junction for precise and fast control of the media dispensed to the cells (the left inset shows 3 typical flow regimes: 100/0, 50/50, and 0/100, respectively) and mother machine channels for long-term monitoring of growing bacteria (right inset); adapted from [6]. DIMM, dual input Mother Machine. (TIF) [file pbio.3000952.s002.tif]

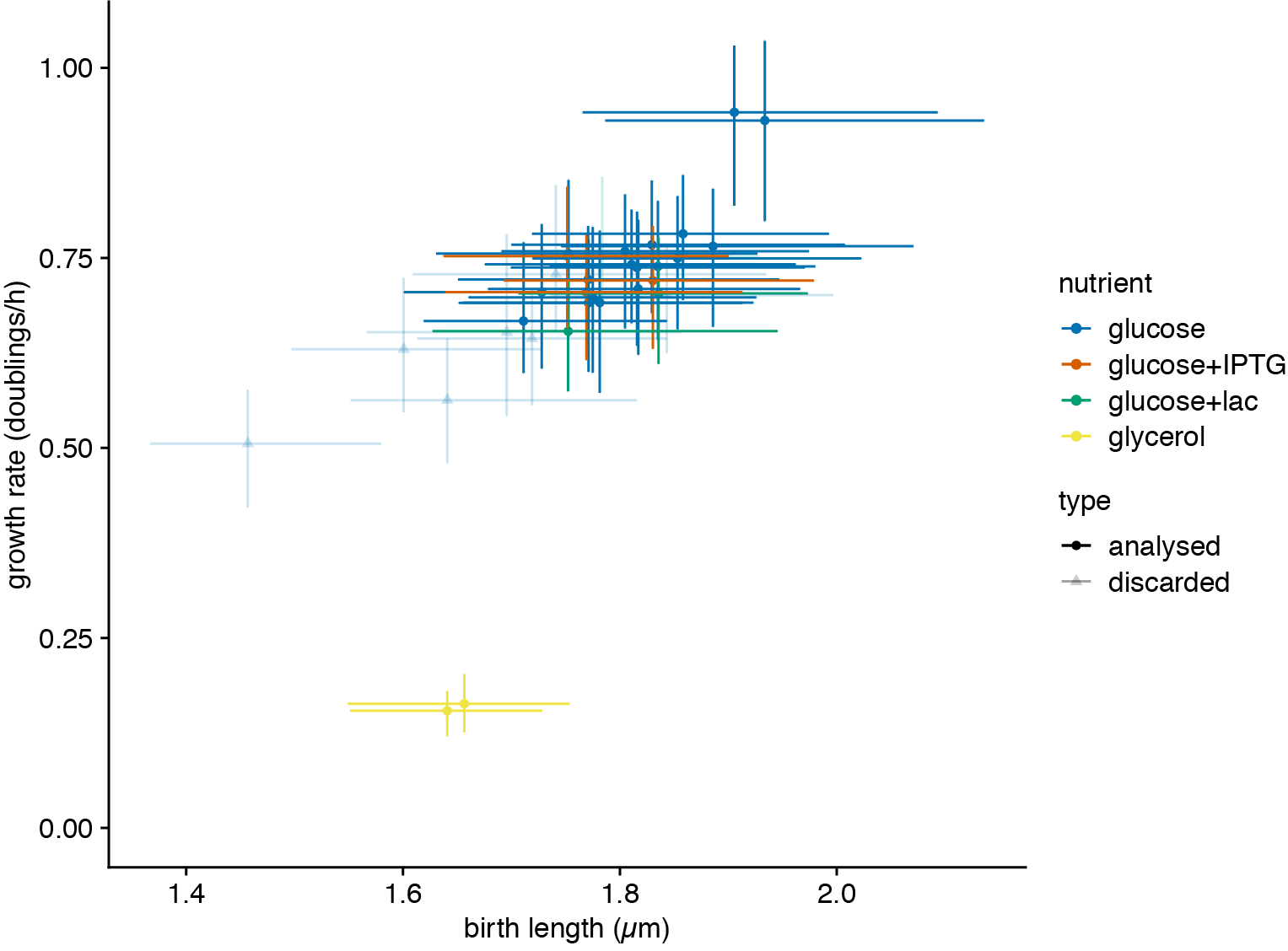

Supplement: S2 Fig — For each experiment, the medians and 95% posterior intervals are plotted of both growth rate and cell length at birth. Note that lower illumination during experiments with low lactose concentration leads to higher growth rate. (TIF) [file pbio.3000952.s003.tif]

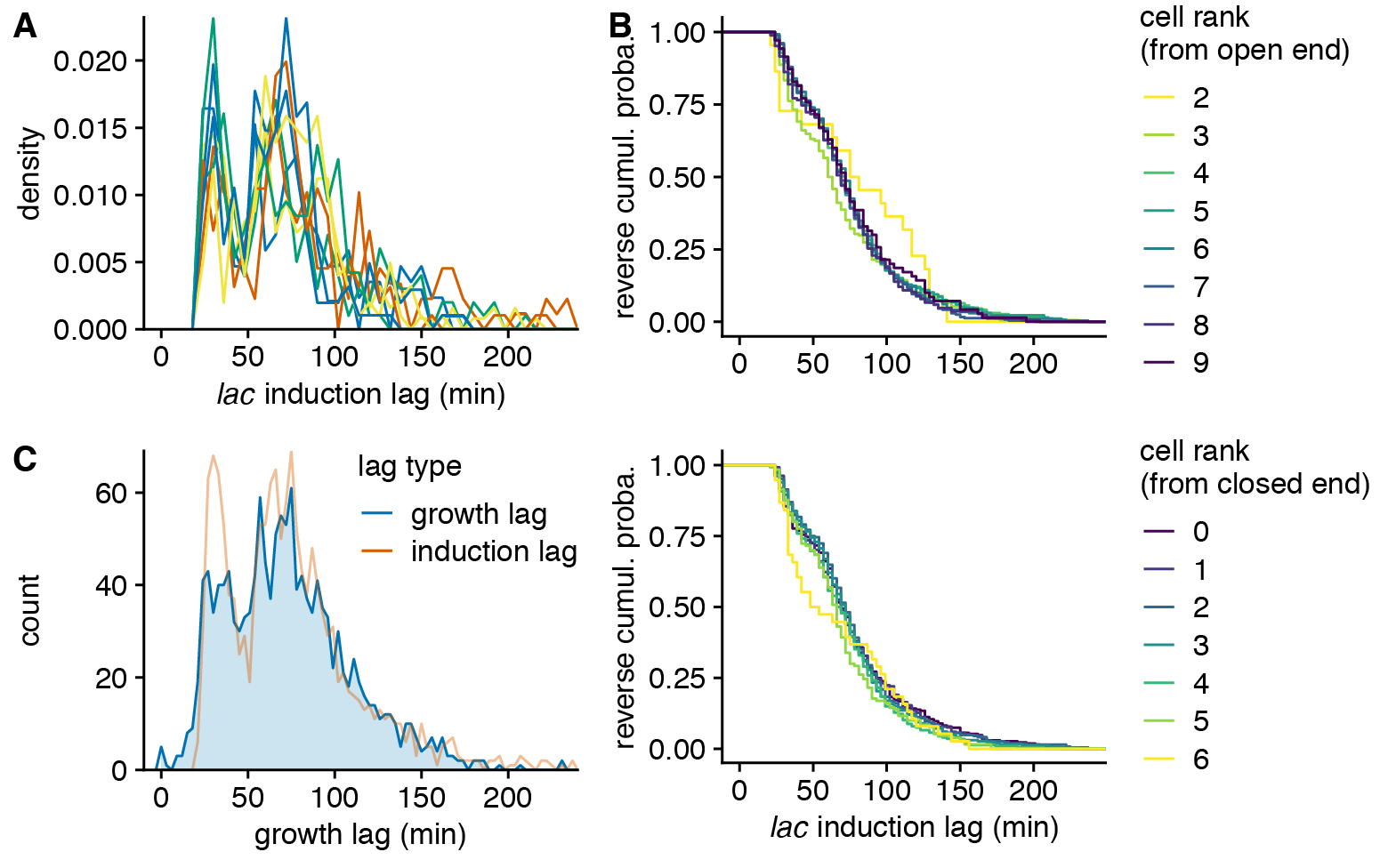

Supplement: S3 Fig — (A) Distribution of induction lags for the lac operon in naive cells, colored per day. Due to the limited sample size in each experiment, the bin width was increased to 6 min. Note that the bimodality of lac induction lag distributions is a robust feature. (B) Distribution of induction lags for the lac operon in naive cells, stratified per position in the growth channels. Position is indicated as cell rank, counted from the cell closest to the channel open end in the upper panel and closest to the closed end in the lower panel. Note that cells close to the open end tend to exit the channel before inducing their lac operon; hence, the distributions are noisier due to smaller sample size. (C) Distribution of growth lags in naive cells. The bin width is the same as the experimental acquisition frequency (3 min). The corresponding distribution of lac induction lags (Fig 1C) is shown for comparison. Note that the distribution of growth lags shows a less marked bimodality, which might result from a combination of less accurate estimation of the growth lag and additional sources of noise being involved in restarting growth once the lac operon is expressed. (TIF) [file pbio.3000952.s004.tif]

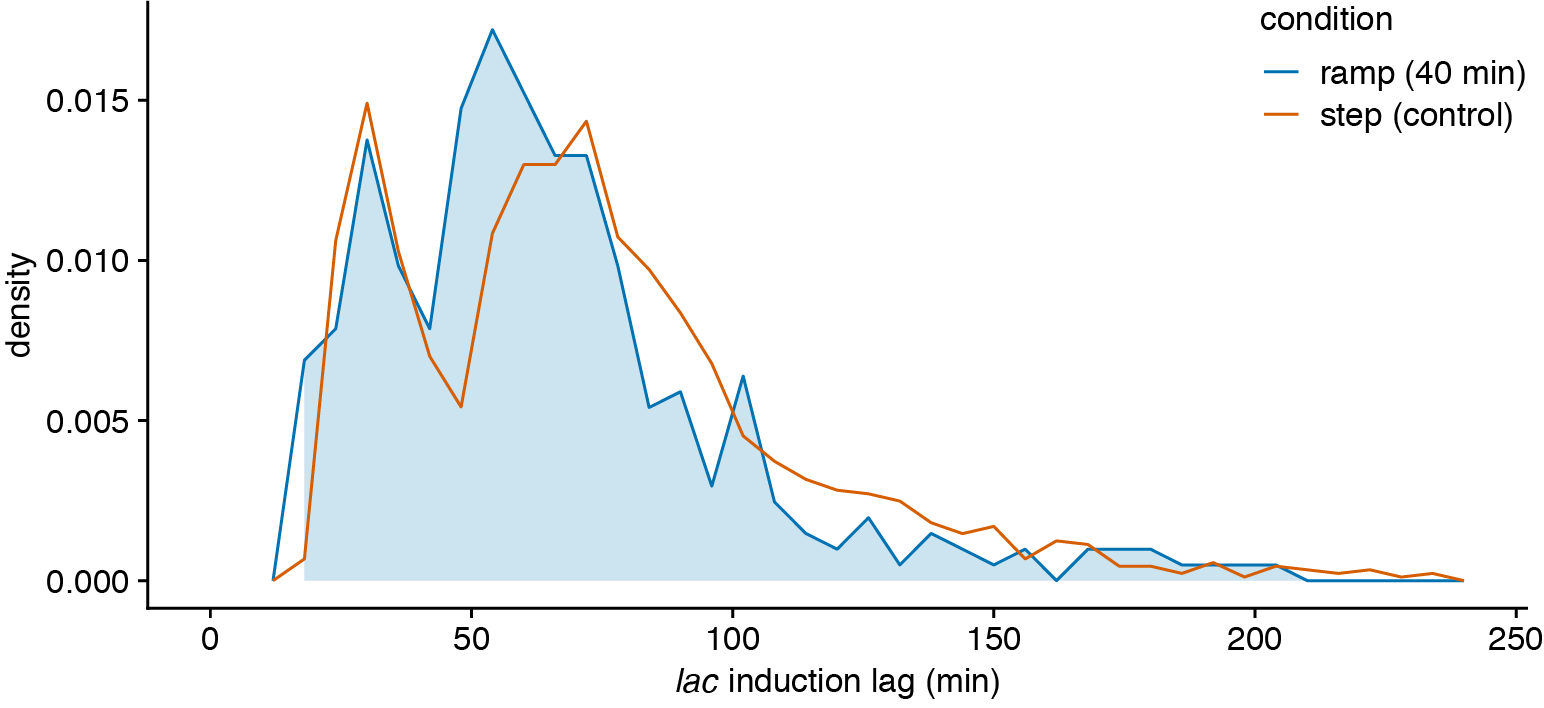

Supplement: S4 Fig — Comparison of the distribution of induction lags for the lac operon in naive cells exposed to a gradual transition from 0.2% glucose to 0.2% lactose over 40 min (2 independent replicates, blue curve) with the distribution of lags under a sudden switch (orange curve, Fig 1C). Note that, since we do not know at what point in the 40 min transition the critical concentrations of glucose/lactose are reached, the lags for each replicate with a gradual transition were offset by a delay that maximized the overlay with the lags under a sudden switch. The fact that the distribution of lags under the gradual transition is almost identical to the distribution under a sudden switch shows that the stochastic single-cell responses remain equally synchronized under the gradual transition, suggesting that there is a common critical concentration of glucose/lactose across all cells. Due to the limited sample size for the gradual transition, the bin width was increased to 6 min. (TIF) [file pbio.3000952.s005.tif]

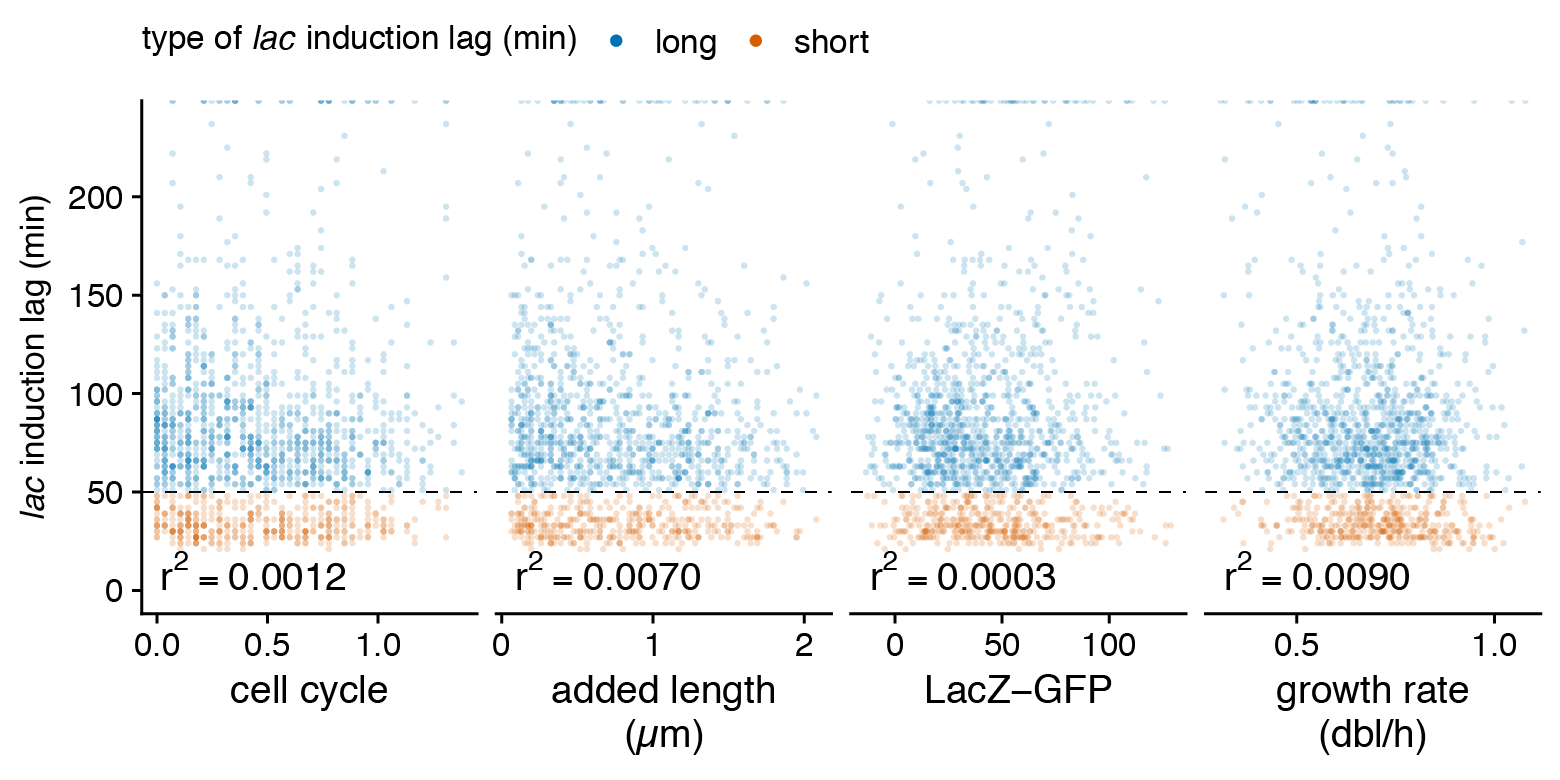

Supplement: S5 Fig — Neither cell cycle progression (measured either as the time since birth normalized to the average division time in this condition or as length added since birth as suggested by the “adder” model of cell cycle control) nor fluorescence at the switch (measured in units of LacZ-GFP molecules) nor growth rate correlate with lac induction time. Note that fast-switching cells are indicated in orange, and slow-switching cells, in blue. GFP, green fluorescent protein. (TIF) [file pbio.3000952.s006.tif]

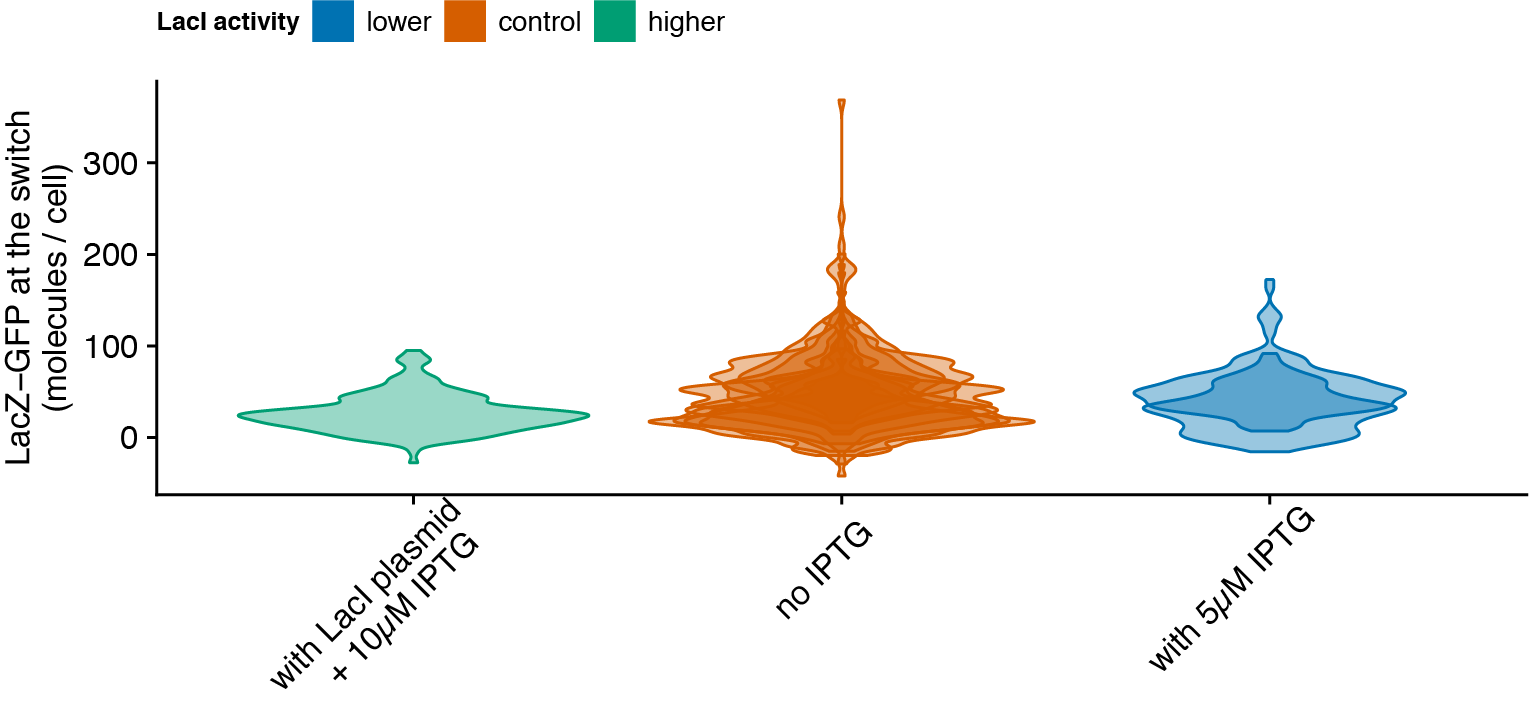

Supplement: S6 Fig — Note that this measurements are imprecise due to relatively large fluctuations in autofluorescence (between cells) and in illumination intensity (between replicates). Although this treatment increases the fraction of fast-switching cells, no detectable change of LacZ-GFP levels can be measured which supports that the increase of basal expression is less than 50 molecules. In comparison, bacteria carry 3000 to 6000 LacZ-GFP molecules at full induction (Fig 1B). GFP, green fluorescent protein. (TIF) [file pbio.3000952.s007.tif]

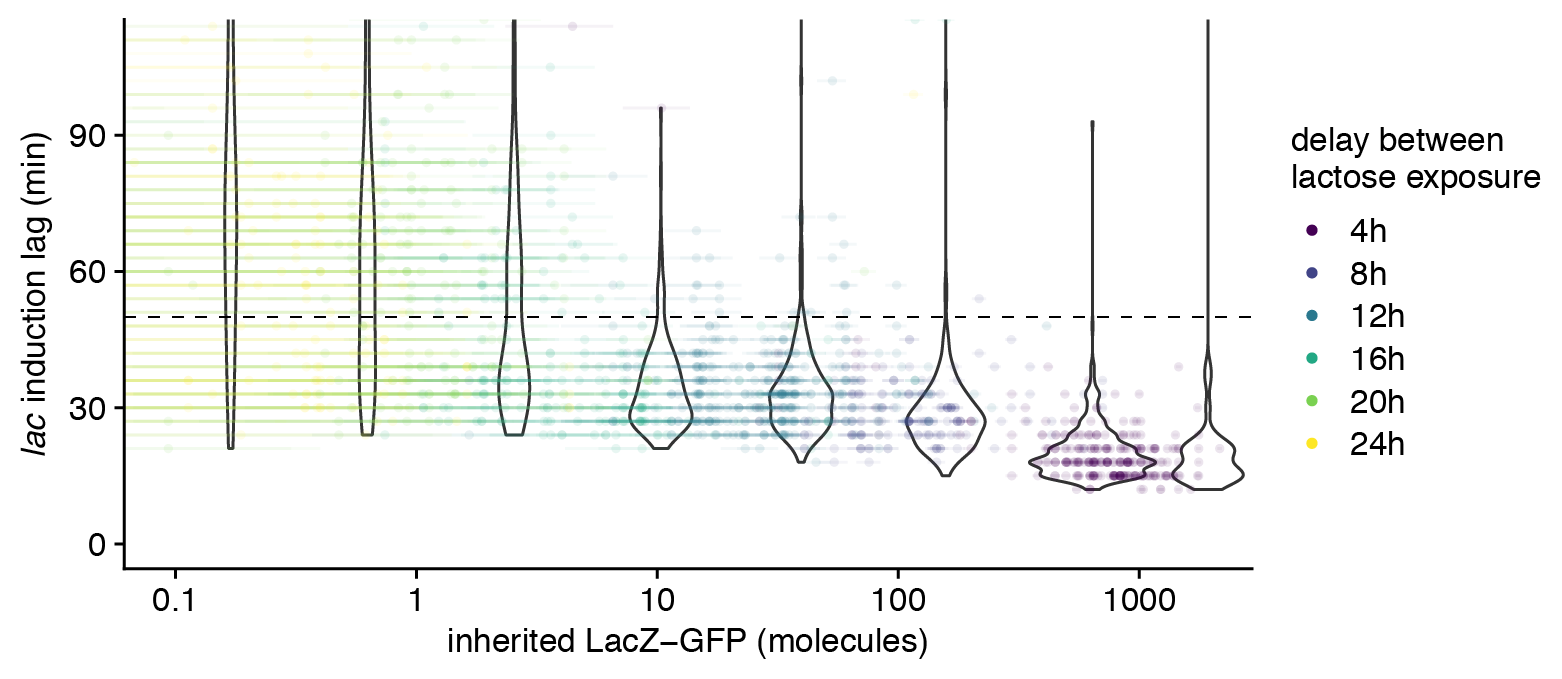

Supplement: S7 Fig — Each dot corresponds to a cell with its estimated lag shown along the vertical axis, its estimated number of remaining LacZ-GFP molecules along the horizontal axis, and its color corresponding to the amount of time the cell spend in glucose between the 2 lactose phases. The dotted line corresponds to the 50-min threshold that separates short from long lags. We stratified the cells into 8 groups depending on their estimated numbers of inherited LacZ-GFP molecules remaining at the second switch, and the violin plots show the distributions of lag times of each group, with its horizontal position centered on the average of the group. Note that long lags only reappear for cells with less than 10 inherited molecules of LacZ-GFP. GFP, green fluorescent protein. (TIF) [file pbio.3000952.s008.tif]

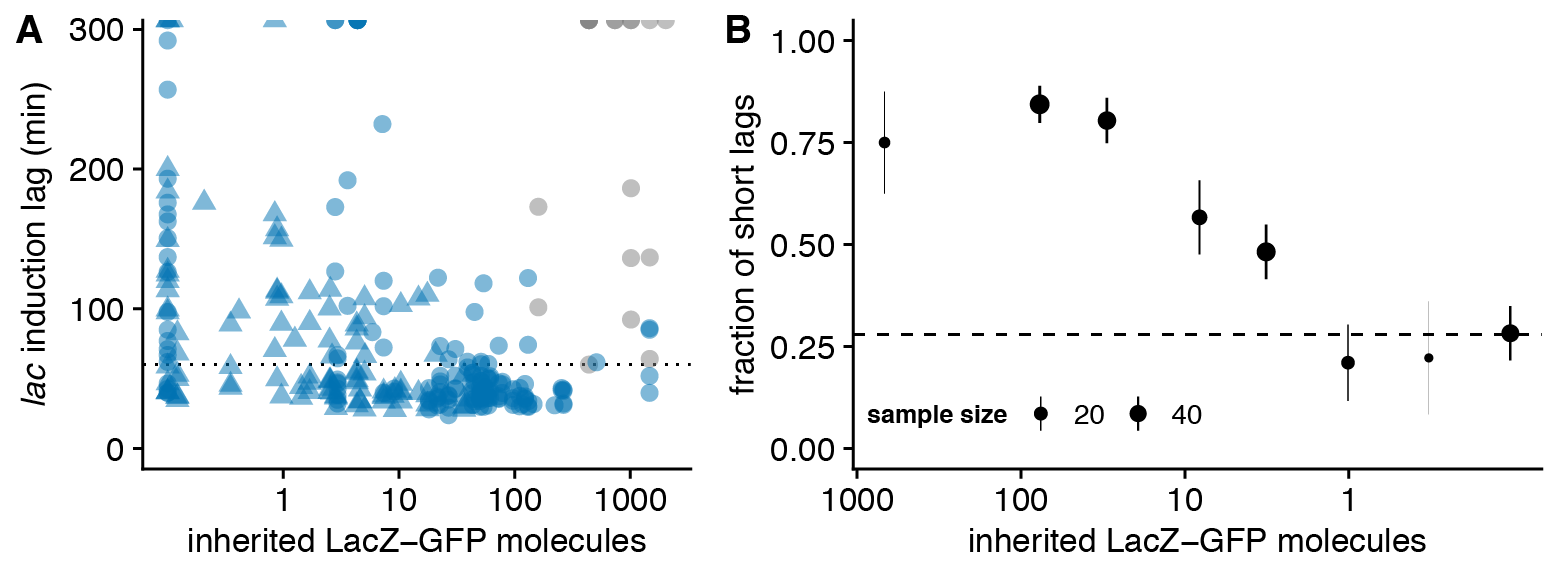

Supplement: S8 Fig — (A) Induction lag as a function of inherited LacZ-GFP (shapes indicate date, and gray dots indicate cells from a lineage with a LacZ-GFP aggregate and are discarded from further analysis). The dotted horizontal line indicates the threshold between short and long lags which is shifted from 50 min (at 0.2% lactose) to 65 min at this lower concentration (0.01%). (B) Fraction of short lags (<65 min) as a function of the estimated number of inherited LacZ-GFP molecules. The dashed horizontal line shows the overall fraction of short lags in naive cells as a reference. GFP, green fluorescent protein. (TIF) [file pbio.3000952.s009.tif]

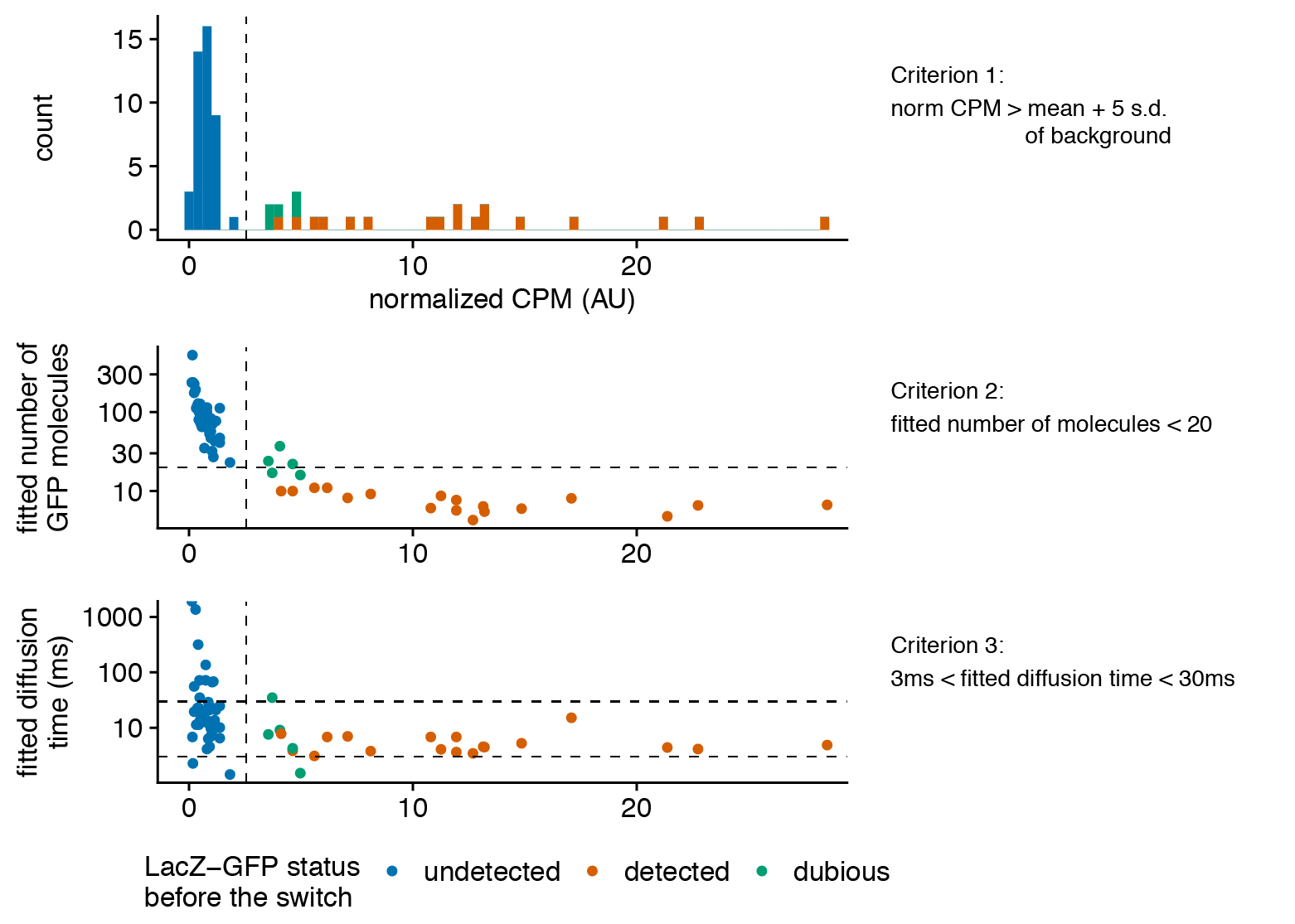

Supplement: S9 Fig — For each cell analyzed with FLCS before the switch in mother machine experiments, 3 criteria must be met concurrently to classify it as GFP positive. Only 5 out of 66 cells where classified as dubious. CPM, counts per molecule; FLIM, fluorescence lifetime imaging; GFP, green fluorescent protein. (TIF) [file pbio.3000952.s010.tif]

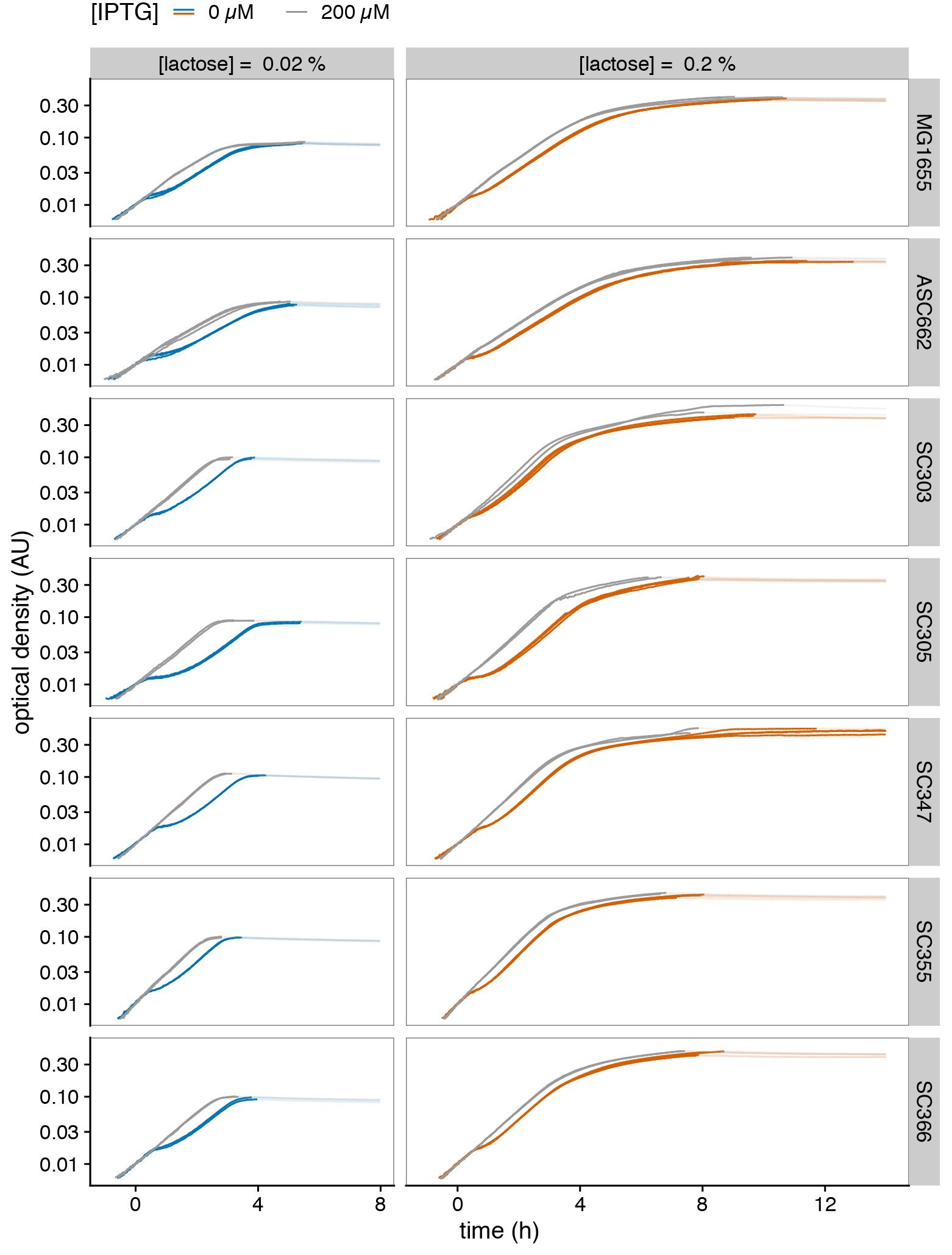

Supplement: S10 Fig — All growth curves used to compute population lags reported in Fig 5B are shown. For each mixture of glucose and lactose (orange lines), the corresponding control with constitutive lac operon expression was obtained by supplementing IPTG (blue lines). Each line corresponds to a biological replicate; and delays were computed for OD below the carrying capacity (solid sections). OD, optical density. (TIF) [file pbio.3000952.s011.tif]

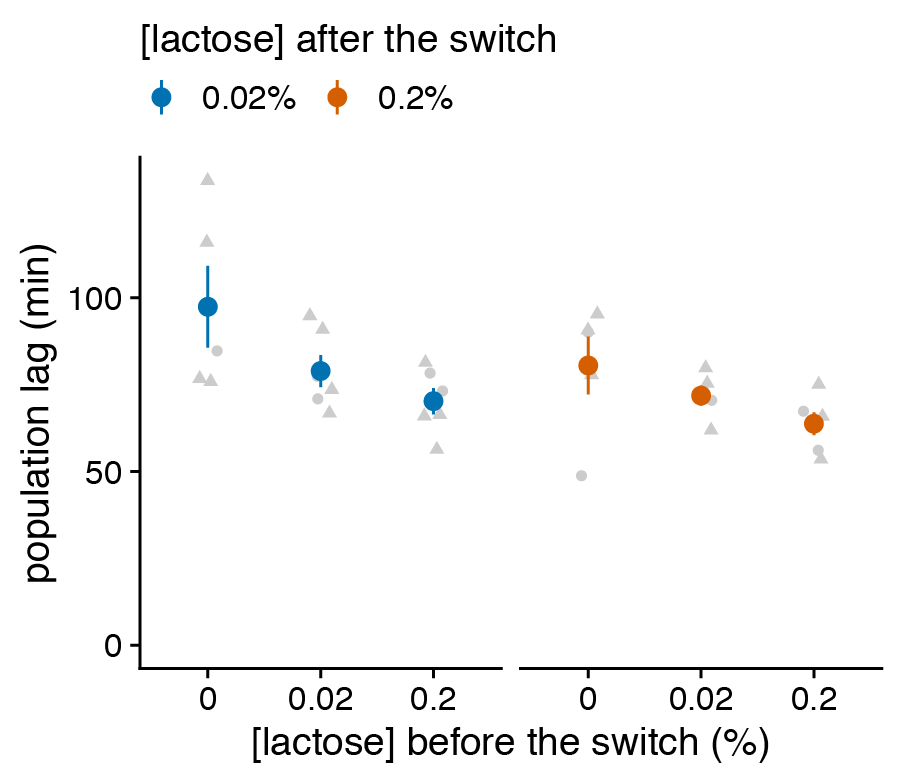

Supplement: S11 Fig — The strain ASC662 was manually transferred from a mixture of glucose (0.005%) and lactose of a given concentration after 4 to 5 h of growth to media with lactose only (OD≈0.1–0.15), at a given concentration. Experiments were performed with 3 different lactose concentrations before the switch and 2 after the switch, for a total of 6 combinations. For each combination, the population lag after the transfer was measured as the delay until OD increased by 0.001 (corresponding to an approximately 10%increase of population size). OD, optical density (TIF) [file pbio.3000952.s012.tif]

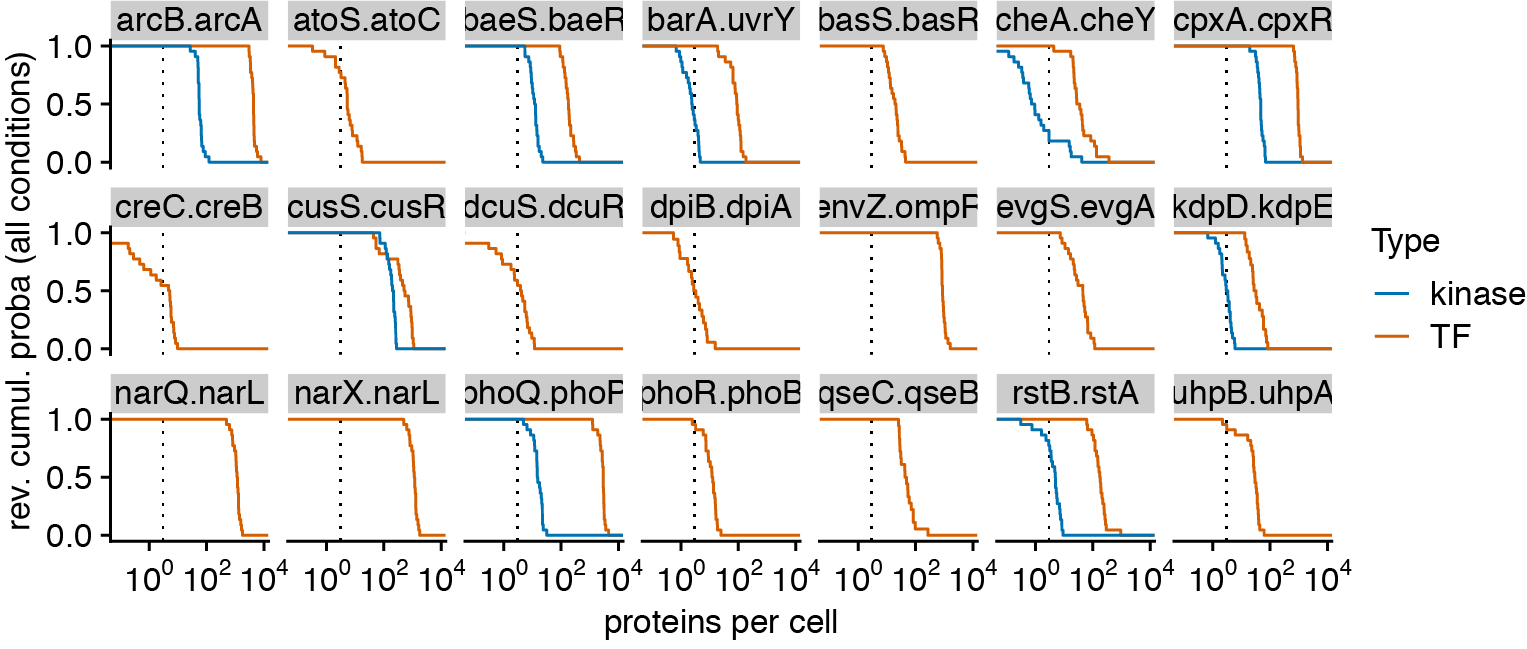

Supplement: S12 Fig — Distributions of expression levels of sensor kinases (blue) and response regulator transcription factors (orange) across 28 conditions as measured using quantitative proteomics (data from [18]) for 21 of the 28 two-component systems annotated on ecocyc.org. Each line shows the reverse cumulative distribution of average number of proteins per cell measured across the 28 conditions. The vertical dashed lines correspond to an average of 3 proteins per cell which is the threshold below which more than 5% of the cells are expected not to have any of the corresponding protein (assuming a Poisson distribution for the abundance of low expressed proteins, i.e., e−3 ≈ 0.05). (TIF) [file pbio.3000952.s013.tif]
