# Supplementary material for: Subpopulations of sensorless bacteria drive fitness in fluctuating environments
Source: PLoS Biol. 2020 Dec 3;18(12):e3000952. doi: 10.1371/journal.pbio.3000952 (PMC7738171; doi:10.1371/journal.pbio.3000952)
Supplement: Attachment — Submitted filename: MoM_lac_induction_rev1-reply.pdf [file pbio.3000952.s015.pdf]

BIOZENTRUM
Universität Basel
The Center for
Molecular Life Sciences

Swiss Institute of
Bioinformatics

University of Basel, Department Biozentrum, Klingelbergstrasse 50/70, 4056 Basel

Roland G Roberts, PhD
Senior Editor
PLOS Biology

Basel, 1 September 2020

Submission of revised manuscript ”Subpopulations of sensorless bacteria drive fitness in fluctuating environments”

Dear Dr Roberts,

We hereby submit a revised version of our manuscript ”Subpopulations of sensorless bacteria drive fitness in fluctuating environments”. We were pleased with the highly constructive comments of the reviewers and have addressed all of them in our revision. Apart from lots of small revisions of the text and added clarifications in response to the comments of the reviewers, we have added three additional experiments to investigate the dependence of the threshold number of pre-existing Lac proteins on the external lactose concentration. These results show that, also at low lactose concentrations, a small number of LacZ and LacY molecules suffices to induce the *lac* operon. This extends our results to show that the single molecule trigger behavior applies over a wide range of external lactose concentrations.

Please find our detailed responses to the reviewers' comments below. We thank the reviewers for their helpful comments and hope that you will now find our manuscript acceptable for publication in PLoS Biology.

Sincerely,

Thomas Julou, PhD

University of Basel
Department Biozentrum
Klingelbergstr. 70
4056 Basel, Switzerland

Thomas Julou
Senior scientist
Tel: +41 (0)61 207 16 21
thomas.julou@unibas.ch

Reviewer #1

Comment 1: *The colors of figure 2B could be changed (these ones are not very easy to distinguish).*

Response 1: We have improved this figure by using a combination of colors and line types to depict delays: like this, the continuous colour scale still captures the continuous nature of the variable while the line types allow a straightforward mapping between any given line and its legend.

Comment 2: *The legend at the bottom of figure 4D could be improved (for instance what gluc to lac full memory means is not clear and the reader has to find the necessary information in the main text).*

Response 2: We agree with the referee and have made some changes to improve the legend. We re-ordered the values to make it easier to read and we replaced ”full memory” by ”no arrest”. In addition, we expanded the legend text for this panel in order to make it easier to understand without referring to the main text.

Comment 3: *I think that fluorescence lifetime imaging is probably classical for physicists but not for biologists and the principle of the technique used here (FLCS) could be explained in more details. I knew the principle of both FLIM and FCS already but I had to struggle a bit to understand what was done here.*

Response 3: We agree that our description of the FLCS technique could have been more clear. For the revision, we better explain in the main text that we employed FLCS because it combines the ability of FLIM to separate spectrally overlapping fluorescent species based on their characteristic lifetime distributions, with the single-molecule sensitivity of FCS. In addition, we have extended the description of the FLCS technique in the Methods section.

. . . .

Reviewer #2

Comment 1: *The authors occasionally mention the role of LacY, and that there is a necessary LacY threshold. However, all their measurements are of LacZ-GFP. It is plausible that LacY and LacZ levels are correlated since they are linked in a polycistronic operon. However, especially given the threshold value of 1 LacZ molecule, it would be important to establish the conditional distribution of LacY given a single molecule of LacZ in single cells. Either the claims of the requirement for LacY should be stated as provisional, or direct measurement of LacY is needed.*

Response 1: We agree that we should have discussed more explicitly the role of LacY in the single-molecule trigger. Although we agree that we only measure LacZ-GFP levels, it has been well established that cells lacking the LacY permease cannot import lactose (*1*, *2*), so that there is little doubt that nonzero LacY levels are required. Nonetheless we agree that in the absence of direct measurement of LacY levels, we cannot yet give a definite estimate of the minimal number of LacY molecules required. However, virtually all cells with more than a handful of LacZ-GFP molecules have short lags (Fig. 2), implying that whenever there are more than a handful of LacZ-GFP molecules, the LacY levels are apparently also always high enough to insure induction. Given that LacY and LacZ are expressed from the same operon, this strongly suggests that the minimal number of LacY molecules required must be low as well. We have clarified these points in the revision (l. 207-212).

Comment 2: *Could the authors provide for the readers a simple back-of-the-envelope calculation, based on the kinetics of lactose transport by LacY and lactose-to-allolactose conversion by LacZ, the number of pre-existing LacY/LacZ molecules that would be nec-*

essary to induce lac operon expression? Related to this, the 1-molecule threshold is of course contingent on external lactose concentration (i.e it applies at high lactose concentration, where the transport machinery is saturated). It must be that, at sufficiently low external lactose, the lag time distribution would change because the threshold LacY/LacZ level would change. Could the authors comment on this?

Response 2: Answering first the second part of the question, we agree that it is interesting to investigate how the external lactose concentration affects the induction dynamics and the sensitivity threshold. For the revision we have now included results from several additional experiments with 20-fold and 200-fold lower concentrations of external lactose.

- at 0.001% lactose (200x less than our standard concentration), *E. coli* does neither induce its *lac* operon nor grow in our microfluidic device.
- at 0.01% lactose (20x less than our standard lactose concentration), we still observe a bimodal distribution, but the induction time for the short lags becomes a bit longer, i.e. lasting up to 65 min (compared to 50 min at 0.2% lactose). We suspect that this delay is due to the slower import of lactose. However, the dilution experiments indicate that the sensitivity threshold does not change much and is still on the same order of magnitude as for high lactose concentrations (1 to 10 LacZ-GFP proteins). Thus, even at this much lower external lactose concentration a few molecules of LacY and LacZ are enough to initiate the positive feedback and de-repress the lac operon.

We have added these results in the main text (l. 219-230) supported by a supplementary figure (Fig. S8), as well as a section in the methods (2.8).

While we agree that it would be nice to present a simple back-of-the-envelope calculation to confirm that, in the presence of a sufficiently high concentration of external lactose, a few LacZ and LacY molecules would be sufficient to destabilize the repressed state of the *lac* operon, we do not believe such a simple calculation is possible. First, there has been quite some theoretical and simulation work on the positive feedback of the *lac* operon which has made clear that the behavior of this system can be fairly complex. For example, the behavior of the system with the natural inducer lactose is qualitatively different from the behavior with artificial inducers such as IPTG and TMG, because LacZ not only produces the allolactose that inhibits LacI, but also hydrolyses both lactose and allolactose. It thus acts to both produce and degrade allolactose. Consequently, there is still no complete consensus in the literature as to whether true bimodality even exists with the natural inducer lactose, or whether it only exists when the operon is induced by artificial inducers such as IPTG and TMG that are not metabolized. This should already make clear that it is not obvious whether a threshold level of LacY/LacZ even exists on the natural inducer lactose, let alone determine what combination of parameters sets it. Moreover, even if we had such a theory, the threshold level would still depend on a combination of kinetic parameters that are only partially known. These parameters include the dependence of lactose transport on external concentrations of lactose and LacY levels, and the kinetics of both the isomerization and hydrolysis reactions that LacZ catalyses. In addition, we would need parameters for modeling the binding and unbinding of allolactose to LacI, the tetramerization of LacI, as well as the dynamics at the lac promoter, i.e. the stochastic switching between LacI binding, partially binding, and unbinding, and the competition with binding by the RNA polymerase. Finally, we would also need to model the dynamics of *lac* operon expression itself, number of translation events per mRNA, time required for protein folding and maturation, and so on. Even if it were possible to accurately model all these processes, it seems clear that rather than a simple and transparent back-of-the-envelope, this would involve developing and simulating a complex model with many parameters that are only partially known. We feel that this is clearly beyond the scope of this manuscript.

Moreover, while it would have been nice if a back-of-the-envelope calculation would have been possible, we would argue that our experimental results are much more meaningful

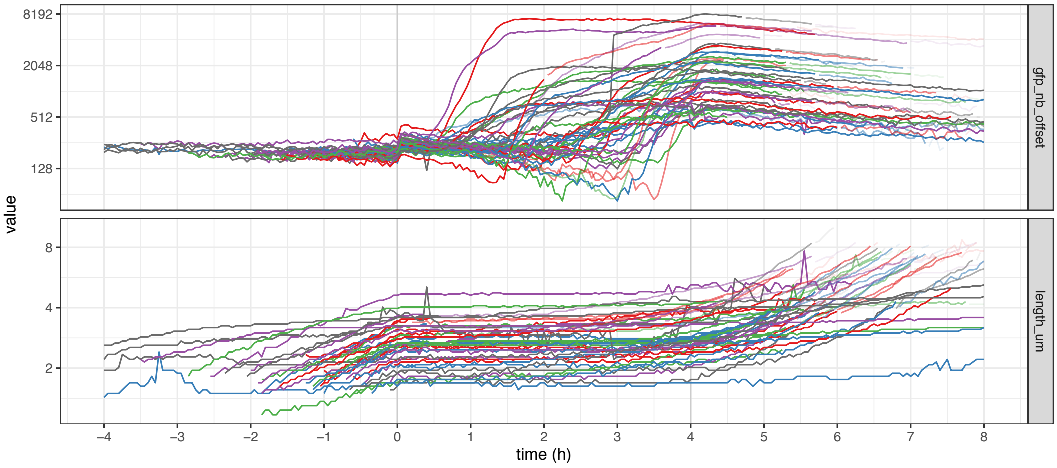

Figure 1: Length and fluorescence traces for cells which meet the criteria for induction of the *lac* operon but do meet the criteria for restarting growth during the lactose period.

since they in fact directly establish that low LacZ levels suffice to induce the lac operon over a fairly large range of concentrations.

Comment 3: *What is the possible origin of the 6% of cells that do not grow even with measurable lac operon expression (line 93)? Given the population heterogeneity, 6% is not obviously associated with being ”rare”.*

Response 3: Replying to this question made us identify a small error in the code used to compute the corresponding proportions: it is in fact not 6% but only 3% of all naive cells measured at the switch which don't regrow on lactose. A systematic look at the corresponding cell traces (see Fig. 1 of this letter) showed that these 3% do not correspond in any case to an homogeneous subpopulation but rather to cells with several categories of behavior: 1. out of 45 cells, 6 don't restart growth even after 4 additional hours in glucose, 2. the majority of the 45 induce their *lac* operon very late during the lactose period so that, even though we detect lac operon induction, they do not meet the criteria for restarting growth before the end of the next switch to glucose, 3. finally approximately 10 cells do induce their *lac* operon early on, but are incapable to restart growth until the next glucose period. It is not clear to us what is the molecular origin of the growth arrest for these cells, but we have noted in various other single-cell projects that transition to new nutrients generally cause a small fraction of the cells to stop growing (either for a long time, or until the next transition).

We updated the text accordingly (and verified that the corresponding mistake in the code did not occur anywhere else). We also updated the corresponding figure panel (1D) since dots corresponding to one observation only were not visible on the initial version.

Although none of these small changes materially affect any of our conclusions, we are grateful that the reviewer's remarks led us to correct this small mistake.

Comment 4: *The authors state that the observed dynamics are unrelated to phase of cell cycle. However, since the pre-existing LacZ threshold is of order 1 molecule, there must be cases in which a cell has a short lag and starts to grow, leading to dilution and partitioning of LacZ, in which one of the daughters again receives no LacZ. I understand this is expected to be rare, but how rare? Have any such trajectories been observed in the existing data?*

Response 4: Although we cannot exclude that such events can happen, there are good

reasons to assume that they are extremely unlikely. Since cellular autofluorescence is as high as several dozens of GFP molecules in each cell and *autofluorescence fluctuates by ± 20 GFP molecules from cell to cell* (Fig. 2A), we can only unambiguously detect LacZ-GFP when there are already on the order of 100 molecules in the cell. It is precisely around this time that we observe cells to resume growth (Fig. 1D). Thus, our data strongly suggests that growth doesn't restart until approximately 100 LacZ-GFP molecules are present. Consequently, by the time that the cell has grown enough to divide, there must be more than 100 LacZ-GFP molecules in the cell, and it is extremely unlikely that one daughter would by chance end up without any LacZ-GFP.

. . . .

Reviewer #3

Comment 1: *The authors skip over one key aspect of these findings* which *is that the molecular mechanism behind sensorless cells is the fact that the lac promoter is bound by LacI, and it's the fluctuation in this operator state that determines whether cells stop becoming sensorless. We had a paper on this in 2014: Bhogale et al., NAR, and there are several other papers that might be mentioned. In our 2014 paper we came to the following conclusion for the lac-operon: Fluctuations in the operator state are the rate-limiting fluctuations for the transition to the induced state. It is a combination of two chance events; (i) the repressor unbinding completely from its binding sites on the lac-operon and (ii) the operator sites of the lac-system staying free of repressors for a time sufficient to produce enough importers for positive feedback to kick in and take the cell into the induced state. I don't like to have to ask the authors to cite one of my own papers, and perhaps there are good reasons not to cite this and other related work by other groups (and I would like to hear them then), but this angle seems pretty relevant to the current study as it is these noisy fluctuations that eventually drive LacZ and LacY production making cells being able to sense and respond (growth) to extracellular lactose.*

Response 1: We agree with the reviewer that we should have added remarks about promoter state fluctuations. In fact, it was so obvious to us that fluctuations in the state of the *lac* promoter ultimately drive the expression of Lac proteins in the repressed state, and thus also exit from this state, that it simply didn't occur to us to mention it explicitly. We have now added a discussion of this point and included the suggested reference to both the Introduction and Results sections (l. 42 and 214). We hope that the current version reads better in this regard.

Comment 2: *Ideally (but not necessary), you would like to put the LacZ-GFP in these non-lab E.coli strains and see if again there is correlation in lag time and lacZ-GFP expression.*

Response 2: We agree with the reviewer that a direct measurement of the induction lag in microfluidics for natural isolates would be a relevant extension of our investigations here. However, two important experimental hurdles are not to be underappreciated.

- Natural isolate strains are typically sticking more to surfaces, hence using them in microfluidic experiments is more challenging and impossible for some strains.
- Our fluorescent reporter for *lac* expression is a scar-less integration of GFP*mut2* between lacZ and lacY which costs more time and efforts to construct than most classical reporters. In addition, we anticipate that using other types of reporters which are easier to obtain might bring in various caveats: for instance, adding another copy of the *lac* promoter to use as a transcriptional reporter would *double* the number of lacI binding sites and hence change the promoter activity by effectively titrating its transcription factor.

For these reasons, we feel that it is out of the scope of this study to directly measure the induction lag of natural isolates in microfluidics.

Comment 3: *L294-305 and Fig. 6. Here the authors attempt to extrapolate analysis from the lac operon to two-component systems present. This last section needs to be expanded and better explained. I get why it's here, it's nice to try to expand past the lac operon, but it could be written more clear. Particularly the figure caption, and how the figure is discussed in the text. Probability of what?*

Response 3: We have rewritten the corresponding section (l. 328-333) and figure caption to make our argument easier to understand.

Comment 4: *L73, is this a promoter fusion or a translational fusion to the enzyme? Clarify here (later it becomes clear it's a translational fusion). Is this fusion protein still fully functional? Are there different lag times in the parental strain without the lacZ-GFP fusion? One would expect it's probably a bit less functional thereby increasing the proportion of* long *lag times (or the GFP might stabilize the protein and its the other way around).*

Response 4: We have edited this (now l. 80) to state explicitly that we use a translational fusion already at this point in the text. Regarding whether this fusion is fully functional, we note that it was reported as such in the article where it was published originally (*3*). Because we were also concerned about possible interactions between LacZ and GFP, we measured the growth rates of ASC662 and of its parental strain MG1655 in M9 minimal medium supplemented with 0.2% glucose and with 0.2% lactose. We noted a small effect on growth rate (≈ 10% decrease) in lactose but not in glucose, suggesting that the translational fusion slightly reduces the catalytic activity of LacZ. Nonetheless, we do not believe that this small change can meaningfully impact the results on the induction dynamics and have other experimental results to support this. For example, in a follow-up of this project, we have measured the distribution of induction lags for another strain which features a transcriptional fusion where lacA is replaced by gfp*mut2*, and the distribution of lags of this strain was still bimodal with the threshold between short and long lags still close to 50 min.

. . . .

Minor comments

- *Line 147: "surprisingly long lags" – The sentence is confusing. I assume the authors are referring to the duration of the intervening period, not the growth lag.*
- *L80 LacZ-GFP; is this the same construct as described in L73?*
- *L209, CRP acronym first time mentioned.*
- *L238, instead of using 'retarded' perhaps better use delayed?*
- *L252, first use of DIMM abbreviation.*
- *L268 & L269 where–>were*

All minor comments have been taken into account in the revised version of the manuscript (note that "CRP" refers to the name of the transcription factor protein in this case, which is indeed an acronym but not used as such) and we thank the reviewers again for their helpful comments that have led to clear improvements of our manuscript.

References

(*1*) Flagg, J. L., and Wilson, T. H. (1976). *lacY* Mutant of *Escherichia coli* with Altered Physiology of Lactose Induction. *Journal of Bacteriology 128*, 701–707.

(*2*) Eames, M., and Kortemme, T. (2012). Cost-Benefit Tradeoffs in Engineered Lac Operons. *Science (New York, N.Y.) 336*, 911–915.

(*3*) Kiviet, D. J., Nghe, P., Walker, N., Boulineau, S., Sunderlikova, V., and Tans, S. J. (2014). Stochasticity of Metabolism and Growth at the Single-Cell Level. *Nature 514*, 376–379.
