# Supplementary material for: Subpopulations of sensorless bacteria drive fitness in fluctuating environments
Source: PLoS Biol. 2020 Dec 3;18(12):e3000952. doi: 10.1371/journal.pbio.3000952 (PMC7738171; doi:10.1371/journal.pbio.3000952)
Supplement: Attachment — Submitted filename: MoM_lacInduction_rev2-reply.pdf [file pbio.3000952.s016.pdf]

Reviewer #3

Comment 1: *One paper that appeared online during the revision of this work is Moreno-Gamez et al in PNAS (https://pubmed.ncbi.nlm.nih.gov/32669426/), which seems very relevant to the current study and the authors might want to include a few lines on how this work connects (or not) to their work.*

Response 1: We understand that, because the article of Moreno-Gamez et al. is concerned with the distribution of single-cell lags in response to lactose, and the way natural selection may have shaped this distribution, it is natural for the reviewer to assume that this work may be relevant to our work. However, we feel that it is extremely unlikely that the evolutionary scenario put forward in this article is relevant for our study, as we explain below.

The main connection to our work is the question of why broad distributions of single-cell lags are observed. In our study, this concerns the subpopulation of sensorless cells with long lags, which significantly affects the population lag and thus fitness. Since the sensory circuit acts as a single-molecule trigger, even a tiny decrease in repression is enough to drastically reduce the fraction of sensorless cells, and thus to improve fitness. This raises the question as to why natural selection has not simply acted to avoid the occurrence of substantial subpopulations of sensorless cells. As explained in the discussion of our paper, this suggests to us that there must either be a cost for even very low expression of the sensor, or that resource constraints preclude cells from expressing all of the many sensory systems at nonzero expression simultaneously.

In contrast, Moreno-Gamez et al. propose that wide distributions of single-cell lags may reflect a bet hedging strategy to deal with a trade-off between growth and survival. In their scenario, Moreno-Gamez et al. propose that short lags are directly selected against, which requires a number of assumptions whose plausibility is questionable in our opinion.

First, the authors assume that fast growing cells are *generally* less likely to survive stress than growth-arrested cells. However, it is currently not clear at all whether such a general association exists. Of course there are stressors that are known to mainly affect growing cells (such as the beta-lactams that the authors use in their study) but we see no reason why there might not be just as many stresses occurring in the wild that affect growth-arrested cells more.

Second, Moreno-Gamez et al. then imagine cells exposed to changing conditions in which the appearance of a new nutrient in the environment is sometimes accompanied by a stress that causes cells that recommence growth to die. In addition, it is assumed that this stress lasts only for a limited amount of time, and disappears while the nutrient is still available, such that cells with long lags can successfully recommence growth and consume the nutrient. That is, a selective environment is envisaged where short lags are explicitly selected against and long lags are beneficial. Although the authors can of course easily set up such a scenario in the lab, we do not see at all why such a specific scenario is likely to occur frequently in the wild.

In order for this scenario to explain the occurrence of sensorless cells and long lags in our system, one would have to assume that the appearance of lactose in the environment is not infrequently accompanied by a stress that kills cells that recommence growth. Moreover, one would have to assume that this stress lasts for a very specific period of time. That is, it must last for more than 30 minutes (because otherwise even cells with short lags won't be affected) but less than 3-4 hours (because otherwise all cells will recommence growth). It is simply not plausible at all to us that this very specific scenario is the reason natural selection has maintained a large fraction of cells with zero expression of the *lac* operon.

We could of course discuss in our paper why we think it is questionable whether the sce-

nario presented by Moreno-Gamez et al. is plausible in general, and very implausible for the system that we study, but we do not think such a discussion is really helpful or constructive.
